# Supplementary material for: Deletion of the Imprinted Phlda2 Gene Increases Placental Passive Permeability in the Mouse
Source: Genes (Basel). 2021 Apr 25;12(5):639. doi: 10.3390/genes12050639 (PMC8146920; doi:10.3390/genes12050639)
Supplement: Supplementary file 1 [file genes-12-00639-s001.zip › genes-1163291-supplementary.pdf]

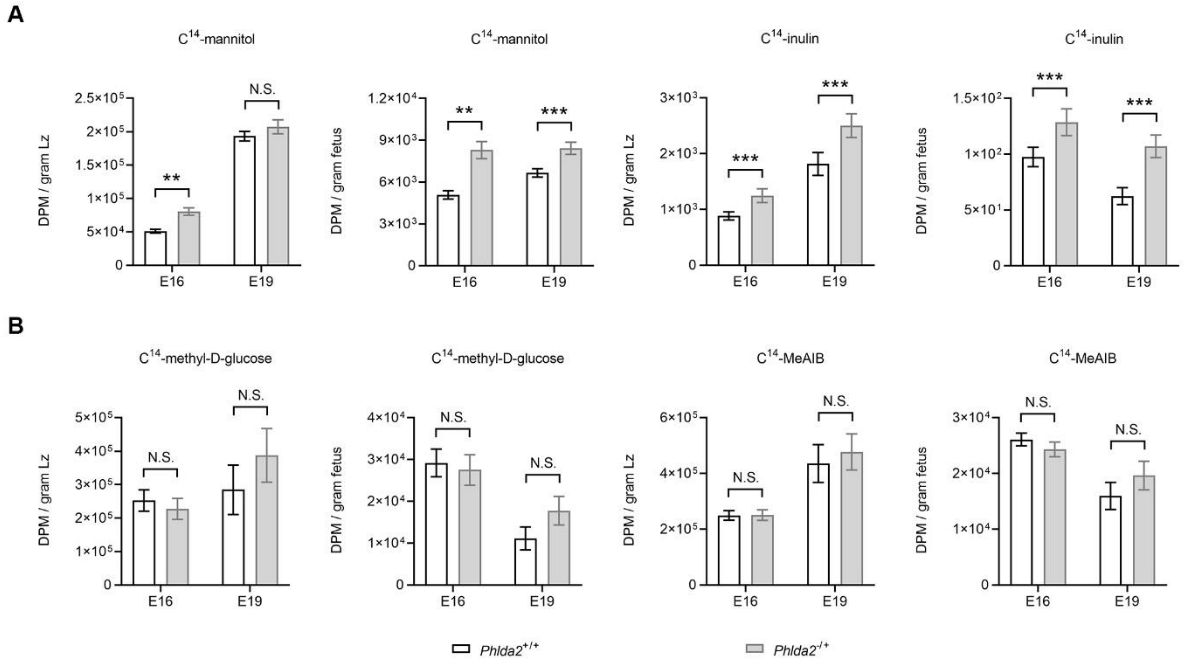

Figure S1. Placental transfer of (**A**) passive permeability markers (^{14}C-mannitol and ^{14}C-inulin) and (**B**) facilitated and active transport markers (^{14}C-methyl-D-glucose and ^{14}C-MeAIB, respectively) in *Phlda2*$^{-/+}$, expressed either as DPM (disintegrations per minute) per gram of labyrinthine zone (Lz) or as DPM per gram of fetus at two gestational ages (E16 and E19). E16: ^{14}C-mannitol, n = 12 *Phlda2*$^{+/+}$ and n = 15 *Phlda2*$^{-/+}$ from 4 litters; ^{14}C-inulin, n = 31 *Phlda2*$^{+/+}$ and n = 32 *Phlda2*$^{-/+}$ from 8 litters; ^{14}C-methyl-D-glucose, *n* = 20 *Phlda2*$^{+/+}$ and *n* = 17 *Phlda2*$^{-/+}$ from 6 litters; ^{14}C-MeAIB, *n* = 16 *Phlda2*$^{+/+}$ and n = 17 *Phlda2*$^{-/+}$ from 5 litters. E19: ^{14}C-mannitol, n = 26 *Phlda2*$^{+/+}$ and n = 35 *Phlda2*$^{-/+}$ from 9 litters; ^{14}C-inulin, n = 22 *Phlda2*$^{+/+}$ and n = 18 *Phlda2*$^{-/+}$ from 5 litters; ^{14}C-methyl-D-glucose, n = 18 *Phlda2*$^{+/+}$ and n = 20 *Phlda2*$^{-/+}$ from 5 litters; ^{14}C-MeAIB, n = 48 *Phlda2*$^{+/+}$ and n = 42 *Phlda2*$^{-/+}$ from 13 litters. Bars indicate standard error of the mean (SE). **, $P < 0.01$; ***, $P < 0.001$; N.S. – non-significant.
